# Androgen Deprivation Therapy and the Risk for Inguinal Hernia: An Observational Nested Case Control Study

**DOI:** 10.1177/15579883211058606

**Published:** 2021-12-17

**Authors:** Maria Hermann, Hanna Vikman, Pär Stattin, Asmatullah Katawazai, Ove Gustafsson, Johan Styrke, Gabriel Sandblom

**Affiliations:** 1 Department of Clinical Sciences, Intervention and Technology (CLINTEC), Karolinska Institute, Stockholm, Sweden; 2 Centre for Digestive Diseases, Karolinska University Hospital, Stockholm, Sweden; 3 Department of Surgical Sciences, Uppsala University Hospital, Uppsala, Sweden; 4 Department of Surgery, Örebro University Hospital, Örebro, Sweden; 5 Department of Surgical and Perioperative Sciences, Urology and Andrology, Umeå University Hospital, Umeå, Sweden; 6 Department of Clinical Science and Education Södersjukhuset, Karolinska Institute, Stockholm, Sweden; 7 Department of Surgery, Södersjukhuset, Stockholm, Sweden

**Keywords:** androgen deprivation therapy, hernia, prostate cancer, inguinal hernia, sex hormones

## Abstract

It has been suggested that hypogonadism increases the risk for inguinal hernia (IH). The aim of this study was to investigate any association between androgen deprivation therapy (ADT) for prostate cancer and increased risk for IH. The study population in this population-based nested case-control study was based on data from the Prostate Cancer Database Sweden. The cohort included all men with prostate cancer who had not received curative treatment. Men who had been diagnosed or had undergone IH repair (*n* = 1,324) were cases and controls, where not diagnosed, nor operated on for IH, matched only on birth year (*n* = 13,240). Conditional multivariate logistic regression models were used to assess any temporal association between ADT and IH, adjusting for marital status, education level, prostate cancer risk category, Charlson Comorbidity Index, ADT, time since prostate cancer diagnosis, and primary prostate cancer treatment. Odds ratio (OR) for diagnosis/repair of IH 0 to 1 year from start of ADT was 0.5 (95% confidence interval [CI] = [0.38, 0.68]); between 1 and 3 years after, the OR was 0.35 (95% CI = [0.26, 0.47]); between 3 and 5 years after, the OR was 0.39 (95% CI = [0.26, 0.56]); between 5 and 7 years after, the OR was 0.6 (95% CI = [0.41, 0.97]); and >9 years after, the OR was 3.68 (95% CI = [2.45, 5.53]). The marked increase in OR for IH after 9 years of ADT supports the hypothesis that low testosterone levels increase the risk for IH. The low risk for IH during the first 8 years on ADT is likely caused by selection of men with advanced cancer unlikely to be diagnosed or treated for IH.

More than 25% of men are diagnosed with inguinal hernia (IH) during their lifetime (Burcharth et al., 2013; HerniaSurge Group, 2018). Although IH is a condition with low morbidity, it can be life-threatening if bowel incarcerates and strangulates. IH repair is the most common general surgical procedure in the world; the pathogenesis of IH, however, is poorly understood. Known risk factors for IH are heredity, male gender (8–10 times more common than in women), age above 70 years, altered connective tissue metabolism, history of radical prostatectomy, smoking, and obesity (Burcharth et al., 2013; Henriksen et al., 2011; Ruhl & Everhart, 2007; HerniaSurge Group, 2018).

Previous studies have reported that men with IH have altered tissue composition in the inguinal region with more fibrotic tissue and atrophic muscles, compared with men without IH (Amato et al., 2012). Due to their impact on connective tissue metabolism, sex hormones may play a crucial role in the development of IH. Most of the estrogen in males is formed by aromatase conversion of testosterone in skeletal muscle and adipose tissue. There is an age-dependent decrease in testosterone levels in men that in turn leads to a fall in serum estrogen levels. It has been suggested that these changes together with an increase in the level of aromatase may cause a weakening of the lower abdominal wall (Vermeulen et al., 2002). This has been reported in a study on a murine model, where an elevated aromatase level per se causes tissue changes in the abdominal wall (Zhao et al., 2018).

Prostate cancer (PCa) is the most common cancer among men in the world. Growth of PCa is stimulated by the presence of testosterone and therefore treated with androgen deprivation therapy (ADT) when there is no intention to cure the disease. There are two types of medical ADT: injections of gonadotropin-releasing hormone (GnRH) analog or antagonist, and oral anti-androgen (AA). Serum testosterone ranges from 270 to 1,070 ng/dL in the healthy male population (Barrett-Connor, 2005). Treatment with GnRH decreases s-testosterone levels below ≤40 ng/dL (Bolton & Lynch, 2018), that is, levels of castration. Treatment with AA, on the contrary, acts in a different way and blocks the androgen receptors present in the prostate, leaving the levels of s-testosterone intact (Osguthorpe & Hagler, 2011). During the study period, GnRH commonly used had the same impact on s-testosterone (Bolton & Lynch, 2018).

The aim of this nested case-control study was to evaluate whether men on ADT for PCa have a higher risk for developing IH than men with PCa but without ADT. Data from Swedish population-based health care registers and demographic databases were used, providing comprehensive data on cancer characteristics, use of ADT, and diagnosis and repair of IH. This information was used to explore whether there are differences regarding type of ADT, PCa risk category, and to estimate time to IH development.

## Method

### Study Population

Data were obtained from the Prostate Cancer Database Sweden (PCBaSe; Hagel et al., 2009; Van Hemelrijck et al., 2016), which is linked to the National Prostate Cancer Register (NPCR; capture rate >98% of all men diagnosed with PCa in Sweden) and 11 other Swedish health care registers and demographic databases, including the Prescribed Drug Register, the Longitudinal Integrated Database for Health Insurance and Labour Market Studies (LISA) providing socioeconomic and demographic information, the National Patient Register, which includes statistics on diseases and surgical treatment, and the Cause of Death Register. Inclusion criteria were diagnosis of PCa, no previous curative intended treatment for PCa (surgery or radiation). Previous curative intended treatment for PCa (surgery or radiation) was considered as exclusion criterion. Men diagnosed or treated for IH were identified in the National Patient Register using their respective code numbers in the International Classification of Diseases–Version 10 (ICD-10; see the appendix) and considered as cases. Some cases had a diagnosis code only, some had a diagnosis code plus a code for hernia repair, and some cases had a code for hernia repair only. The cases were matched with 10 controls (1:10) only on birth year. The study was approved by an Ethics Committee (Table 1).

**Table 1.** Inclusion and Exclusion Criteria for the Cohort.

	Cases	Controls
Inclusion	• Prostate cancer diagnosis • No previous curative intended treatment for prostate cancer • Diagnosis or repair of inguinal hernia	• Prostate cancer diagnosis • No previous curative intended treatment for prostate cancer
Exclusion	• Previous curative intended treatment for prostate cancer	• Previous curative intended treatment for prostate cancer

### Study Measures

The cohort that served as a base in this nested case-control study consisted of all men registered in PCBaSe4 with PCa between January 1, 2008 and December 31, 2016 without having received treatment with curative intent (*n* = 30,823). Of these, 1,324 men considered that cases were identified in the National Patient Register. These men had been diagnosed or treated for IH according to ICD codes (see the appendix) prior to December 31, 2017. Men with IH diagnosed or repaired within 6 months prior the start of ADT were not included. For each case, 10 men matched for year of birth were selected from the same cohort as the controls in accordance with the principles of a nested case-control study (Hennessy et al., 1999). No matching for other variables was done. Time elapsed between beginning of anti-androgen therapy (ADT) and IH diagnosis, and/or repair was categorized as follows: no ADT, GnRH analog treatment < 1 year, GnRH 1 to 3 years, GnRH 3 to 5 years, GnRH 5 to 7 years, GnRH 7 to 9 years, GnRH >9 years, and AA monotherapy.

ICD codes for the diagnosis IH and/or repair were used as outcome measure (K40.0-K41.9, JAB00-JAB80, JAC10-JAC40; see the appendix).

The Charlson Comorbidity Index (CCI) was calculated using discharge diagnoses in the National Patient Register during the 10-year period prior to PCa diagnosis (Charlson et al., 1987). LISA provided information on marital status, single or married, and education level divided into three categories: low, <10 years; intermediate, 10 to 12 years; and high, >12 years. The PCBaSe provided data on PCa risk category according to a modification of the guidelines of the National Comprehensive Cancer Network (NCCN; Mohler et al., 2019; Table 2). Time elapsed since diagnosis of PCa and form of primary treatment were also collected from PCBaSe. The latter was categorized as watchful waiting (WW), where ADT is started if symptoms progress, active surveillance (AS) defined as prostate specific antigen (PSA) <10 and age <70 years, and other (mix of AS and WW).

**Table 2.** Prostate Cancer Risk Categories From the PCBaSe Based on a Modification of the Guidelines of the NCCN.

Risk categories	Definition
Localized prostate cancer	
Low risk	T1-2, Gleason score 2 to 6, and PSA <10 ng/mL
Intermediate risk	T1-2, Gleason score 7, and/or PSA 10 to <20 ng/mL
High risk	T3 and/or Gleason score 8 to 10 and/or PSA 20 to <50 ng/mL
Regional metastasis	T4 and/or N1 and/or PSA 50 to <100 ng/mL in the absence of distant metastases (M0 or Mx)
Distant metastases	M1 and/or PSA ≥100 ng/mL

*Note.* PCBaSe = Prostate Cancer Database Sweden; NCCN = National Comprehensive Cancer Network; PSA = Prostate Specific Antigen; Mx = No information/ investigation about metastasis.

### Statistical Analysis

Conditional multivariate logistic regression models were used to calculate odds ratios (ORs). The exposure variable was defined as ADT, and the endpoint was risk for diagnosis or repair of IH. All ORs were adjusted for marital status, education level, PCa risk category, CCI, total time on ADT, type of ADT time from PCa diagnosis, and primary PCa treatment. Total time of ADT was divided into eight groups: no ADT, GnRh <1 year, GnRh 1 to 3 years, GnRh 3 to 5 years, GnRh 5 to 7 years, GnRh 7 to 9 years, GnRh >9 years, and AA as monotherapy. The time interval of 3 years was chosen to give a narrow estimate of time of ADT treatment and development of IH as reasonable.

## Results

Distributions of marital status and education level among men with a diagnosis of IH or repair were similar to matched controls (Table 3).

**Table 3.** Baseline Characteristics of Non-Curative Treated Prostate Cancer Cases Diagnosed 2008 to 2016 and Their Matched Controls.

	Cases	Controls
	*n* = 1,324	*n* = 13,240
Age, median (Q1, Q3)	76 (70, 82)	76 (70, 82)
Marital status, *n* (%)		
Married	932 (70%)	8,721 (66%)
Not married	392 (30%)	4,519 (34%)
Education level, *n* (%)		
Low	475 (36%)	4,484 (34%)
Intermediate	324 (24%)	3,487 (26%)
High	525 (40%)	4,931 (37%)
Missing	0 (0%)	338 (2.6%)
Risk category, *n* (%)		
Low	566 (43%)	4,550 (34%)
Middle	349 (26%)	3,119 (24%)
High	214 (16%)	2,780 (21%)
Regional metastasis	64 (4.8%)	916 (6.9%)
Distant metastases	89 (6.7%)	1,472 (11%)
Missing	42 (3.2%)	403 (3.0%)
CCI, *n* (%)		
0	795 (60%)	6,830 (52%)
1	247 (19%)	2,675 (20%)
2	125 (9.4%)	1,594 (12%)
3+	157 (12%)	2,141 (16%)
ADT, *n* (%)		
No ADT	742 (56%)	6,159 (47%)
GnRH <1 year	65 (4.9%)	1,074 (8.1%)
GnRH 1–3 years	66 (5.0%)	1,614 (12%)
GnRH 3–5 years	40 (3.0%)	865 (6.5%)
GnRH 5–7 years	27 (2.0%)	443 (3.3%)
GnRH 7–9 years	19 (1.4%)	203 (1.5%)
GnRH >9 years	49 (3.7%)	123 (0.9%)
Anti-androgen monotherapy	316 (24%)	2,759 (21%)
Time since PCa diagnosis		
<5 years	968 (73%)	8,862 (67%)
5–9 years	260 (20%)	3,195 (24%)
10–14 years	84 (6.3%)	1,005 (7.6%)
>15 years	12 (0.9%)	178 (1.3%)
Primary treatment		
AS	537 (41%)	4,369 (33%)
WW	295 (22%)	2,715 (21%)
Other	492 (37%)	6,156 (46%)

*Note.* Definitions: PCa risk category—low risk (T1-2, Gleason score 2–6, and PSA <10 ng/mL), intermediate risk (T1-2, Gleason score 7, and/or PSA 10 to <20 ng/mL), and high risk (T3 and/or Gleason score 8–10 and/or PSA 20 to <50 ng/mL); regional metastatic disease—T4 and/or N1 and/or PSA 50 to <100 ng/mL in the absence of distant metastases (M0 or Mx); distant metastases—M1 and/or PSA >100 ng/mL and above. CCI = Charlson Comorbidity Index; ADT = androgen deprivation therapy; GnRH = gonadotropin-releasing hormone; PCa = prostate cancer; AS = active surveillance; WW = watchful waiting.

Men with IH had a lower PCa risk at diagnosis than controls. AS as primary treatment was more frequent among men with IH than controls. Of the men with IH, *n* = 741 (56%) were not prescribed ADT compared with *n* = 6,223 (47%) of the controls. CCI was zero in *n* = 794 (60%) of men with IH compared with *n* = 6,885 (52%) in controls, indicating that men with hernia had less aggressive PCa and better general health.

The OR for hernia after at least 9 years treatment with GnRH was 3.32 (95% confidence interval [CI] = [2.33, 4.73]) in the adjusted logistic regression analysis. On the contrary, ADT with GnRH for up to 7 years or monotherapy with AA was associated with a decreased risk for IH in the unadjusted as well as adjusted models (Table 4).

**Table 4.** Adjusted and Unadjusted ORs for Inguinal Hernia Associated With ADT According to Length of Treatment.

	Unadjusted model		Adjusted model	
	OR	95% CI	OR	95% CI
Marital status				
Married		Reference	1.00	Reference
Not married	0.81	[0.72, 0.92]	0.91	[0.81, 1.04]
Education level				
Low	1.00	Reference	1.00	Reference
Intermediate	0.86	[0.76, 1.03]	0.86	[0.74, 1.00]
High	1.01	[0.87, 1.16]	0.93	[0.81, 1.06]
Missing	NA	NA	NA	NA
Risk category				
Low	1.00	Reference	1.00	Reference
Middle	0.87	[0.75, 1.00]	0.93	[0.79, 1.09]
High	0.58	[0.48, 0.68]	0.75	[0.60, 0.92]
Regional metastasis	0.53	[0.40, 0.69]	0.76	[0.56, 1.04]
Distant metastases	0.47	[0.37, 0.59]	0.86	[0.64, 1.15]
Missing	0.81	[0.58, 1.12]	0.88	[0.62, 1.23]
CCI, *n* (%)				
0	1.00	Reference	1.00	Reference
1	0.78	[0.67, 0.90]	0.81	[0.69, 0.94]
2	0.66	[0.54, 0.80]	0.69	[0.57, 0.85]
3+	0.61	[0.51, 0.74]	0.69	[0.57, 0.83]
ADT				
No ADT	1.00	Reference	1.00	Reference
GnRH <1 year	0.47	[0.36, 0.62]	0.51	[0.38, 0.68]
GnRH 1–3 years	0.31	[0.24, 0.40]	0.35	[0.26, 0.47]
GnRH 3–5 years	0.35	[0.25, 0.48]	0.39	[0.26, 0.56]
GnRH 5–7 years	0.47	[0.31, 0.71]	0.63	[0.41, 0.97]
GnRH 7–9 years	0.75	[0.46, 1.23]	1.07	[0.65, 1.78]
GnRH >9 years	3.32	[2.33, 4.73]	3.68	[2.45, 5.53]
Anti-androgen monotherapy	0.90	[0.78, 1.04]	0.91	[0.77, 1.07]
Time since PCa diagnosis				
<5 years	1.00	Reference	1.00	Reference
5–9 years	0.72	[0.62, 0.84]	0.70	[0.60, 0.81]
10–14 years	0.72	[0.57, 0.92]	0.63	[0.49, 0.82]
>15 years	0.57	[0.31, 1.04]	0.43	[0.23, 0.79]
Primary treatment				
AS	1.00	Reference	1.00	Reference
WW	0.829	[0.54, 0.71]	0.92	[0.77, 1.10]
Other	0.83	[0.70, 0.98]	0.94	[0.79, 1.11]

*Note.* Definitions: PCa risk category—low risk (T1-2, Gleason score 2–6, and PSA <10 ng/mL), intermediate risk (T1-2, Gleason score 7, and/or PSA 10 to <20 ng/mL), and high risk (T3 and/or Gleason score 8–10 and/or PSA 20 to <50 ng/mL); regional metastatic disease—T4 and/or N1 and/or PSA 50 to <100 ng/mL in the absence of distant metastases (M0 or Mx); distant metastases—M1 and/or PSA > 100 ng/mL and above. ADT = androgen deprivation therapy; OR = odds ratio; CI = confidence interval; CCI = Charlson Comorbidity Index; GnRH = gonadotropin-releasing hormone; PCa = prostate cancer; AS = active surveillance; WW = watchful waiting.

Unadjusted OR from conditional logistic regression. Adjusted OR from conditional logistic regression with adjustment for marital status, education level, PCa risk category, CCI, previous ADT treatment, time since prostate cancer diagnosis, and primary treatment.

The cases had more low-risk PCa and more frequently a CCI of zero, indicating a better general health than the controls (Table 3).

## Discussion

In this population-based nested case-control study, men who had been on ADT with GnRH for more than 9 years showed an increased risk for IH, whereas the risk for IH among men treated with AA monotherapy, that is, with no alterations in s-testosterone, was no different to controls. This finding could support the hypothesis that a low androgen level increases the risk for IH. To our knowledge, this is the first study investigating the effect of sex hormones on the development of IH.

The pathogenesis of IH is unclear, complex, and multifactorial, but histological studies have reported findings of more muscle atrophy and fibrosis in the lower abdominal wall especially around the internal inguinal ring in men with IH than among men without IH (Amato et al., 2012).

That the cases had better general health, that is, lower CCI than the controls, may be explained by the fact that more physically active men are more prone to seek medical attention for a benign condition as an IH than frail men with poorer health. Furthermore, physicians may be more alert to diagnose a benign condition like IH in healthier men.

Androgens like testosterone are anabolic and longtime suppression of testosterone with GnRH leads to muscular atrophy. Presence of estrogen stimulates fibroblasts responsible for fibrinization. The disturbed ratio between testosterone and estrogen could be promoting IH development in men treated with GnRH both due to muscle atrophy and elevated fibrinization leading to a weakening of the lower abdominal wall. A study on physiological aging men showed that estrogen levels decrease more slowly than testosterone levels, with a higher estrogen: testosterone ratio for the first 8 years of the study period (Rohrmann et al., 2011). Other studies (Amato et al., 2012; Vermeulen et al., 2002) have reported that aromatase activity increases in older men, causing locally higher production of estrogen in muscle and adipose tissue that stimulates the fibroblasts. It is reasonable to believe that activity increases in men treated with GnRH as well and since the anabolic effect of androgens is absent, which could increase the incidence of IH in men treated with GnRH. This is supported by the fact that men treated with AA monotherapy, with normal testosterone levels, do not have a higher incidence of IH.

It could also be hypothesized that other conditions with increased risk for IH development may also involve changes in hormonal/enzymatic activity. Family clustering may be due to abnormal expression of the aromatase gene. Increased aromatase activity in obese men leads not only to high estrogen levels, that may increase the fibrinization in the lower abdominal wall, but also increased intra-abdominal pressure, a known risk factor for IH.

We believe that the present findings warrant further research into the investigation and treatment of hypogonadism in elderly men to prevent IH formation, especially in cases with recurrent IH and in families with a history of IH.

### Strengths and Limitations

Strengths of this study: The nested case-control design performed on well-defined large population-based cohort with a long complete follow-up.

There are some limitations to this study. One major drawback is that it is a register-based study using retrospective data to indicate the endpoint IH. Even though it has been mandatory to register all inpatients and outpatients in the Swedish National Patient Register since 2001, thus providing nationwide coverage of cases with repaired IH, the primary health care is not covered by the National Patient Register, which could result in a falsely low estimate of the prevalence of IH in the population, but this would apply to the whole cohort, because cases and controls in a nested case-control study are from the same cohort.

Although there were statistically significant associations, we cannot draw definite conclusions regarding the causal relationships. There may be other mechanisms than those described here involved.

We do not have any information about previous IH as a child; a previous study identified a slightly increased risk of IH formation later in life if a male has undergone IH repair in childhood (Sokratous et al., 2019). This increase is so small that we believe that the large cohort will cover for this.

## Conclusion

Longtime treatment, more than 9 years, with ADT with GnRH was associated with an increased risk for IH which indirectly supports the hypothesis that a low androgen level increases the risk for IH.

## Appendix

### International Classification of Diseases–Version 10 (ICD-10) Codes

#### Diagnosis: Inguinal Hernia

K40.0

K40.1

K40.1A

K40.1B

K40.2

K40.2A

K40.2B

K40.3

K40.3A

K40.3B

K40.4K40.4A

K40.4B

K40.9

K41.0

K41.1

K41.2

K41.3

K41.4

K41.9

#### Procedure: Surgery for Inguinal Hernia

JAB30

JAB00

JAB01

JAB04

JAB10

JAB11

JAB14

JAB20

JAB40

JAB41

JAB44

JAB50

JAB51

JAB60

JAB61

JAB70

JAB71

JAB80

JAC10

JAC11

JAC30

JAC40
